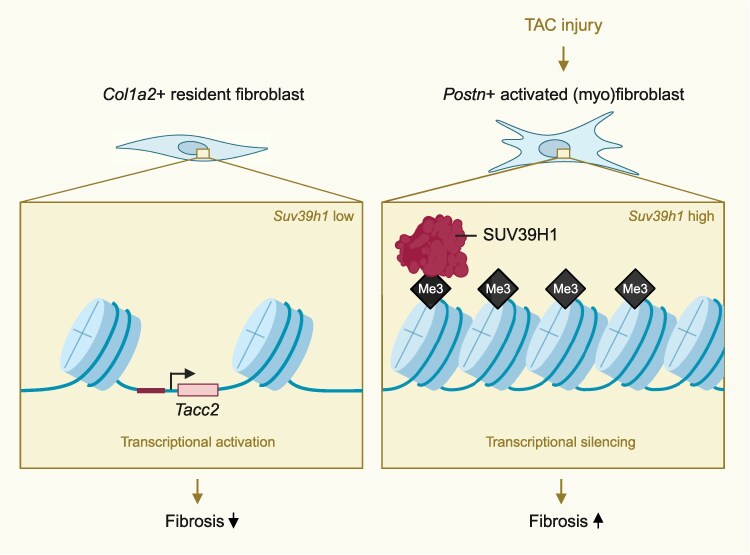# Repressive chromatin regulates fibrotic potential through *Suv39h1* in the heart

**DOI:** 10.1093/cvr/cvag071

**Published:** 2026-03-30

**Authors:** Tim Koopmans, Eva van Rooij

**Affiliations:** Hubrecht Institute, Royal Netherlands Academy of Arts and Sciences (KNAW) and University Medical Center Utrecht, P.O. Box 85164, 3508 AD, Utrecht, Netherlands; Hubrecht Institute, Royal Netherlands Academy of Arts and Sciences (KNAW) and University Medical Center Utrecht, P.O. Box 85164, 3508 AD, Utrecht, Netherlands; Department of Cardiology, University Medical Center Utrecht, Heidelberglaan 100, 3584 CX, Utrecht, Netherlands

**Keywords:** Fibrosis, Fibroblasts, Regeneration, Chromatin, Heart disease

**This editorial refers to ‘Suppressor of variegation 3–9 homolog 1 deficiency attenuates cardiac fibrosis and rescues heart failure through TACC2’, by C. Zhu *et al.*, https://doi.org/10.1093/cvr/cvag066.**

Diverse forms of heart disease frequently converge on a shared, chronic stage of cardiac dysfunction termed heart failure, a complex clinical syndrome characterized by impaired cardiac output, typically accompanied by cardiomyocyte hypertrophy,^1^ reactivation of foetal gene programmes,^2^ and the development of a detrimental fibrotic response.^3^ In heart failure with preserved ejection fraction, diffuse interstitial and perivascular fibrosis substantially increases ventricular stiffness and filling pressures, impairing diastolic relaxation.^3^ In heart failure with reduced ejection fraction, fibrosis more commonly reflects replacement scarring following cardiomyocyte loss, disrupting force transduction and electrical coupling between cardiomyocytes, leading to progressive systolic dysfunction, chamber dilation, and arrhythmias.^3^ Despite considerable efforts, clinical translation of anti-fibrotic therapies has been challenging, and dose-limiting adverse effects underscore the biological complexity and pleiotropy of fibrotic signalling pathways.^4^

In this issue of *Cardiovascular Research*, Zhu *et al*. explore an alternative strategy to mitigate cardiac fibrosis by directly targeting cardiac fibroblasts through modulation of the histone methyltransferase SUV39H1, a key enzyme catalysing histone H3 lysine 9 trimethylation (H3K9me3). Within the broader framework of regenerative medicine, modulation of H3K9me3 represents a chromatin-level intervention with the potential to coordinately regulate large gene networks. SUV39H1 primarily establishes H3K9me3 at constitutive heterochromatin: gene-poor, repeat-rich genomic regions that remain highly condensed during interphase and are frequently associated with the nuclear periphery. These domains consist largely of satellite DNA arranged in tandem arrays, particularly at (peri)centromeric and telomeric regions, but can also be found in intergenic regions. At these sites, H3K9me3 contributes to chromosomal stability, faithful segregation during mitosis, suppression of transposable elements, and maintenance of genome integrity.^5^

In addition to constitutive heterochromatin, a subset of SUV39H1-dependent H3K9me3 marks localizes to facultative, locus-specific heterochromatin. Within this context, H3K9me3 contributes to the repression of lineage-inappropriate genes, directly or through higher-order chromatin topology,^6^ for example, during liver development,^7^ or immune cell differentiation.^8–10^ Building on this framework, Zhu *et al*. demonstrate that in adult cardiac fibroblasts, SUV39H1 occupies and represses promoters of fibrosis-associated genes. Primary cardiac fibroblasts isolated from mice subjected to transverse aortic constriction (TAC) to model heart failure exhibit increased *Suv39h1* expression during myofibroblast activation, which is further enhanced by exposure to pro-fibrotic stimuli such as TGF-β. Genetic ablation or siRNA-mediated knockdown of *Suv39h1* attenuated myofibroblast activation markers and reduced profibrotic gene expression, as well as repressing typical myofibroblast behaviours related to (TGF-β-induced) proliferation, migration, and contractility. Although the authors do not demonstrate changes in H3K9me3 levels following *Suv39h1* manipulation, their findings favour the idea that heterochromatin landscapes are dynamically regulated under fibrotic conditions, a finding that aligns with earlier work, partly from the same lab, implicating *Suv39h1* in hepatic stellate cells^11^ or hepatocytes^12^ with liver fibrosis, or with cardiac fibrosis after ischaemic injury.^13^

To lend credence to these claims, fibroblast-specific deletion of *Suv39h1* using *Col1a2*- or *Postn*-driven Cre lines to target resident and activated fibroblasts respectively, were employed to study fibrotic conditions in mice. Although *Suv39h1* deletion did not prevent TAC-induced cardiomyocyte hypertrophy, which may be expected as the transgene does not directly affect cardiomyocytes, it significantly reduced interstitial fibrosis and myofibroblast accumulation, leading to partial improvement in systolic function 6 weeks after TAC. Conversely, AAV9 was employed to overexpress *Suv39h1* under control of the *Postn* promoter, which, despite tropism for cardiomyocytes, was sufficient to exacerbate fibrotic remodelling. Although some the experiments could benefit from more rigour, these findings reinforce the pathological relevance of *Suv39h1*.

To further define mechanism, the authors performed H3K9me3 CUT&TAG-seq in cultured human primary cardiac fibroblasts following Suv39h1 knockdown. Several promoter regions were identified as SUV39H1-associated. Among 17 putative SUV39H1-repressed regions, the authors decide to focus on TACC2 (transforming acidic coiled-coil containing protein 2). They proposed that under fibrotic conditions, TGF-β promotes recruitment of SUV39H1 to the TACC2 promoter, leading to its repression. In line with their reasoning, TGF-β, but also angiotensin II, and endothelin-1 (other commonly used pro-fibrotic stimulators) reduced TACC2 expression in a SUV39H1-dependent manner. Functional assays revealed that TACC2 overexpression attenuated, whereas its suppression enhanced, myofibroblast activation phenotypes, suggesting that TACC2 acts antagonistically to fibrosis-related responses.

Although a follow-up for TACC2 would have been interesting, the authors decide to instead explore the therapeutic relevance of SUV39H1 by focusing on a small-molecule inhibitor, F5446, reported to inhibit SUV39H1 methyltransferase activity. Although enzyme selectivity and on-target engagement were not directly demonstrated in their system, interestingly, F5446-treated TAC mice displayed modest reductions in hypertrophic parameters in addition to decreased fibrosis and improved function, effects that diverge from the fibroblast-restricted genetic deletion model, and likely owing to its non-specific cellular targeting. An intriguing finding, as it may indicate SUV39H1-dependent roles in non-fibroblast populations, such as cardiomyocytes. These results tie in with earlier work demonstrating a beneficial role for whole-body deletion of *Suv39h1* in adult mice following ischaemic injury to the heart.^13^

Overall, the study introduces a relatively unexplored concept: that repressive histone modifications traditionally associated with constitutive heterochromatin may be therapeutically exploitable in fibrosis, although several mechanistic and technical limitations preclude definitive conclusions. For example, constitutive heterochromatin domains, spanning large repetitive genomic regions essential for nuclear architecture and chromosomal stability, were not systematically examined. Given the fundamental roles of H3K9me3 in genome integrity and lineage stabilization, it remains unclear how sustained SUV39H1 inhibition might affect fibroblast identity, chromosomal stability, or broader transcriptional fidelity over time. Furthermore, protein validation in human diseased cardiac tissue is lacking, leaving translational relevance to be established. Nevertheless, the authors provide a compelling framework that positions SUV39H1-controlled heterochromatin as a relevant component of cardiac fibrosis. With many questions remaining to be answered, further developments in this area are eagerly awaited.

**Figure:**